# Supplementary figures and images for: Abrupt dietary changes between grass and hay alter faecal microbiota of ponies
Source: PLoS One. 2020 Aug 18;15(8):e0237869. doi: 10.1371/journal.pone.0237869 (PMC7446798; doi:10.1371/journal.pone.0237869)

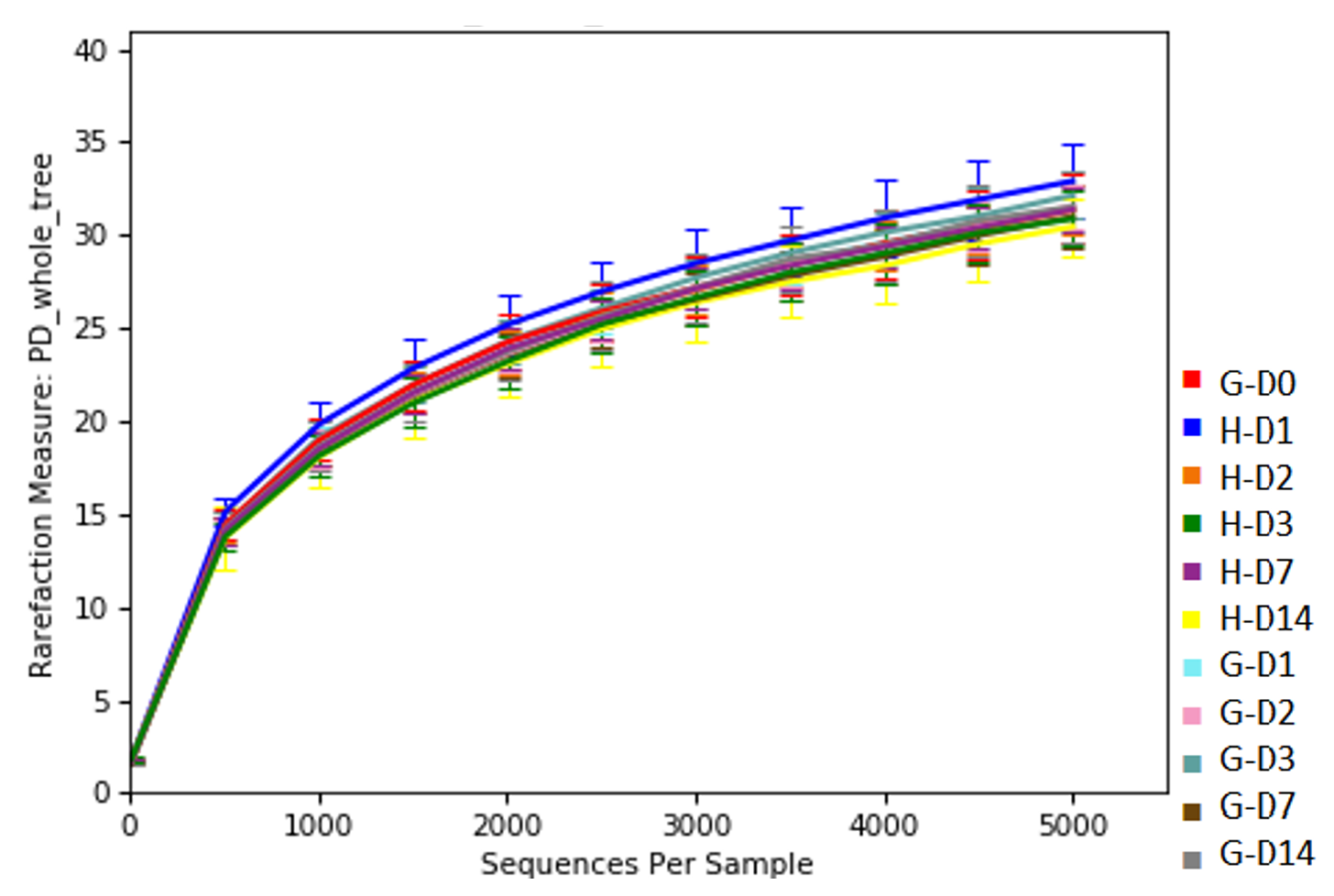

Supplement: S1 Fig — Each curve represents sampling day (D) on grass (G) or hay (H). (TIF) [file pone.0237869.s001.tif]

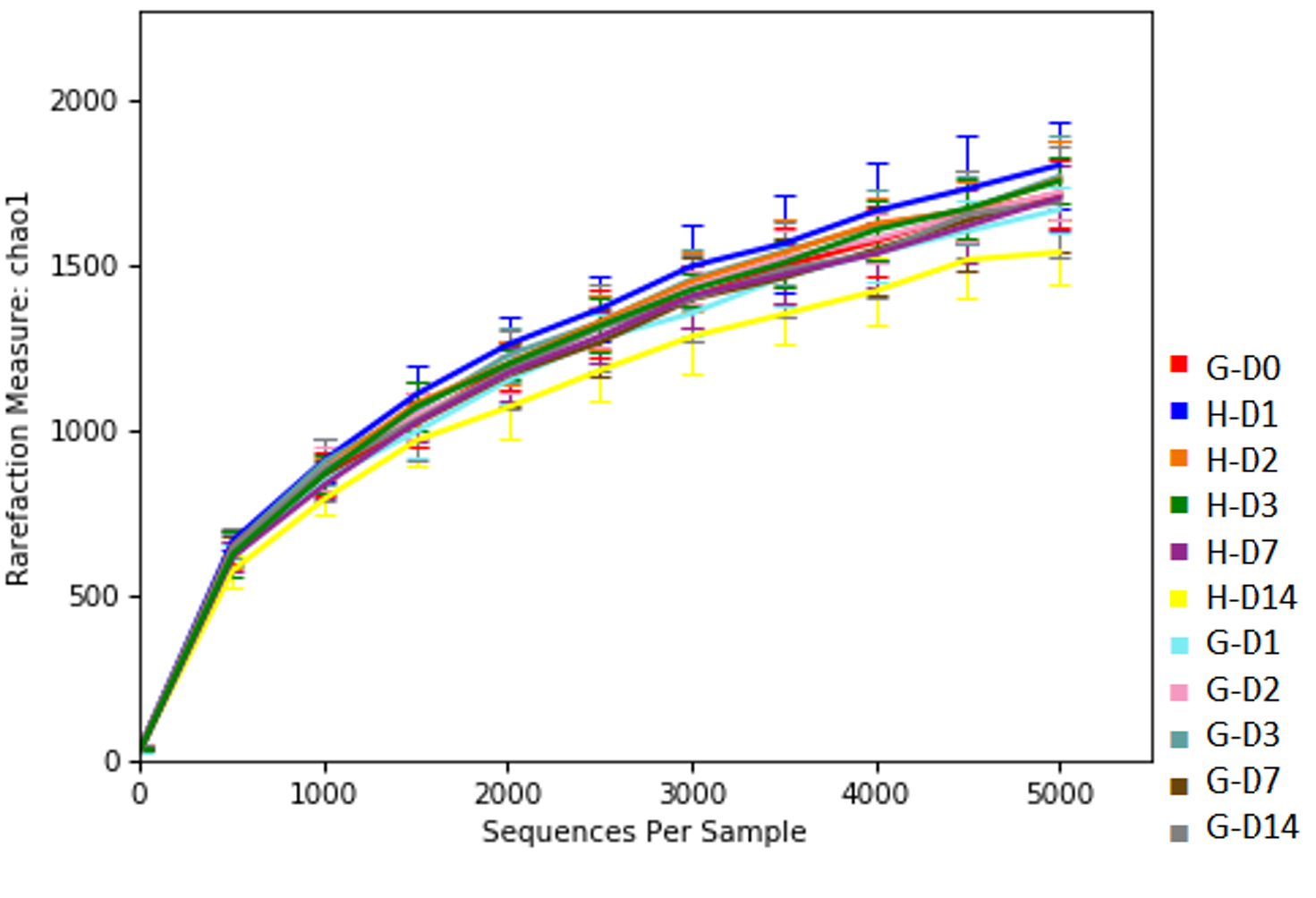

Supplement: S2 Fig — Each curve represents sampling day (D) on grass (G) or hay (H). (TIF) [file pone.0237869.s002.tif]

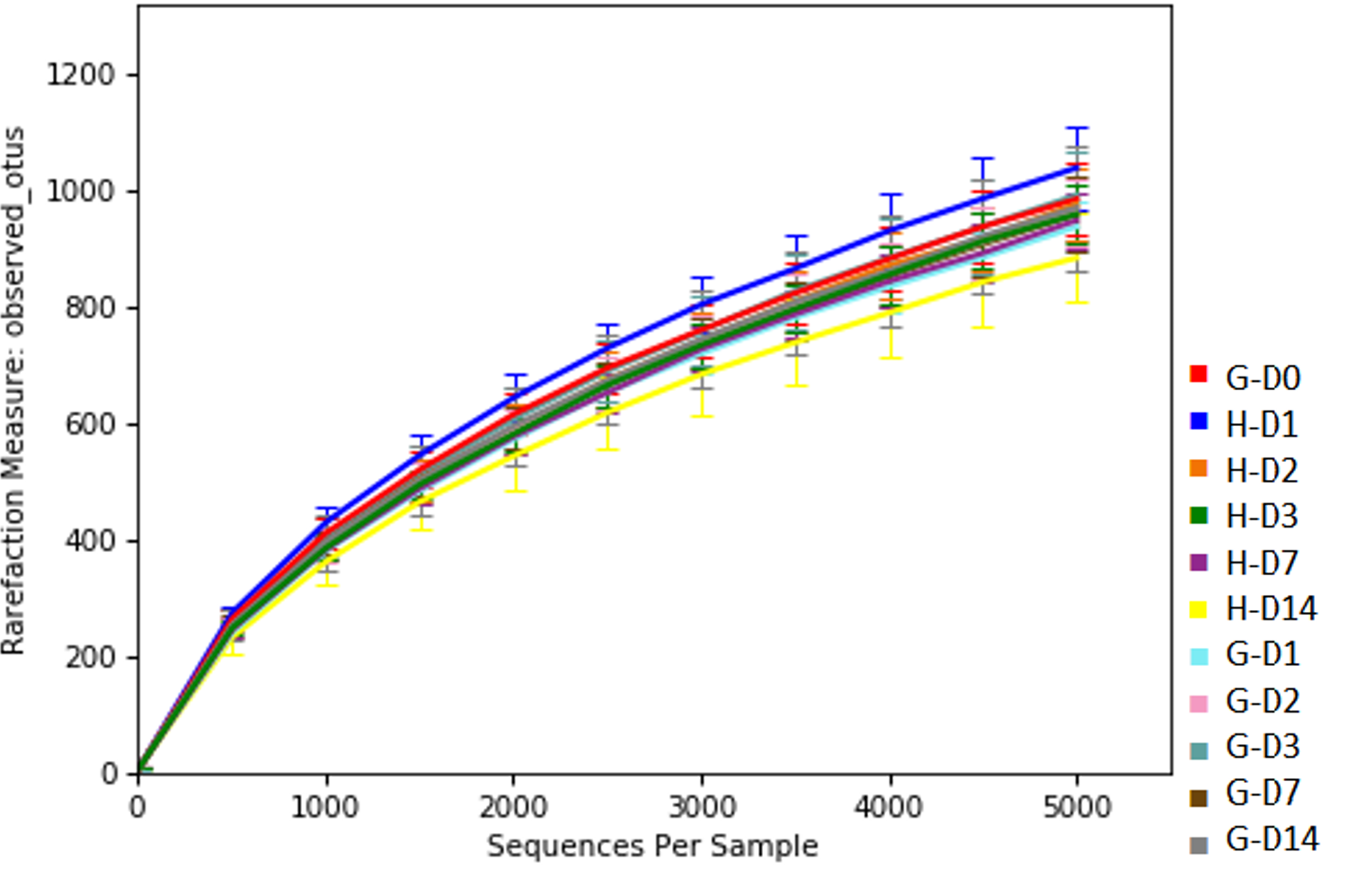

Supplement: S3 Fig — Each curve represents sampling day (D) on grass (G) or hay (H). (TIF) [file pone.0237869.s003.tif]

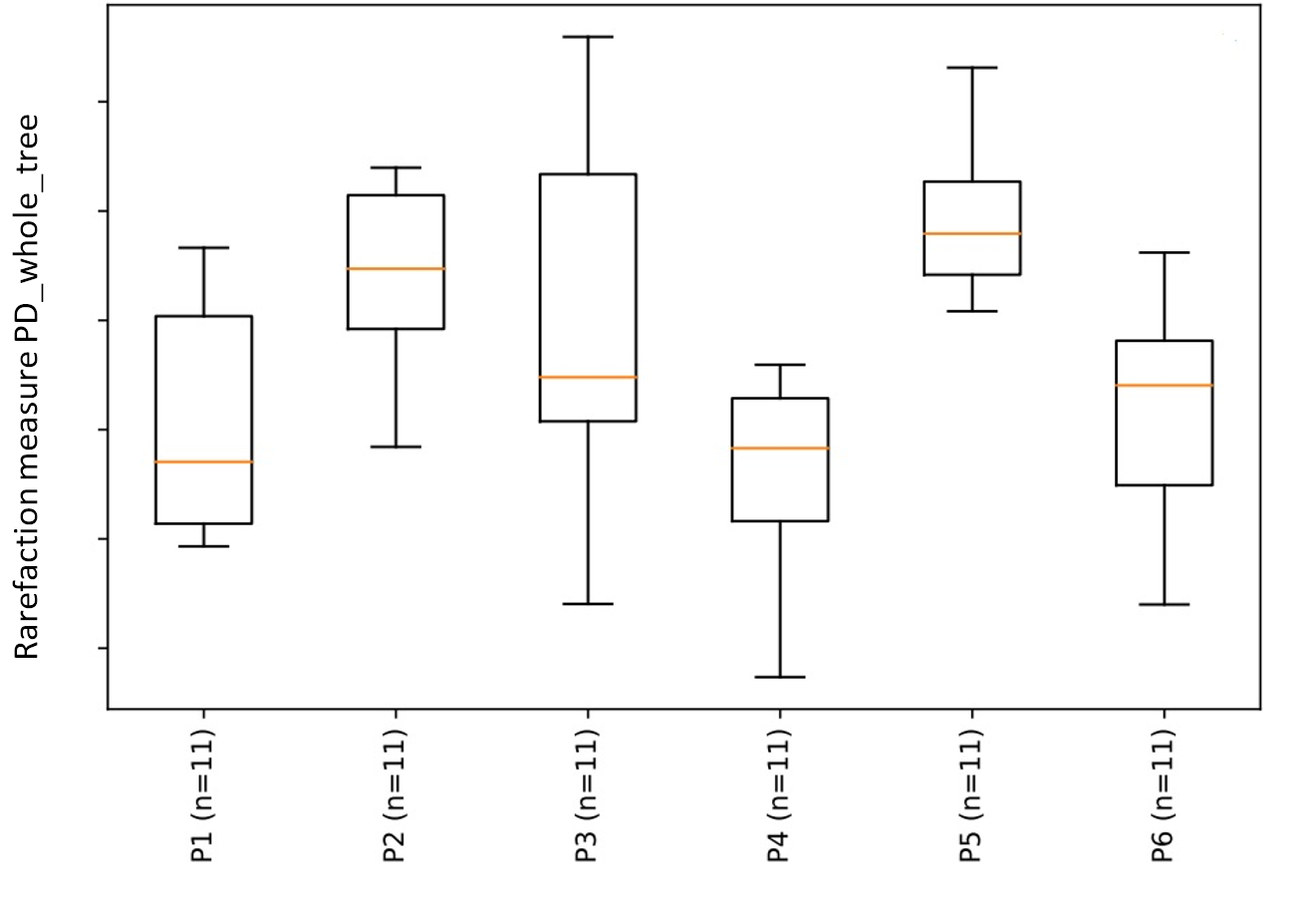

Supplement: S4 Fig — n: number of samples per pony. (TIF) [file pone.0237869.s004.tif]

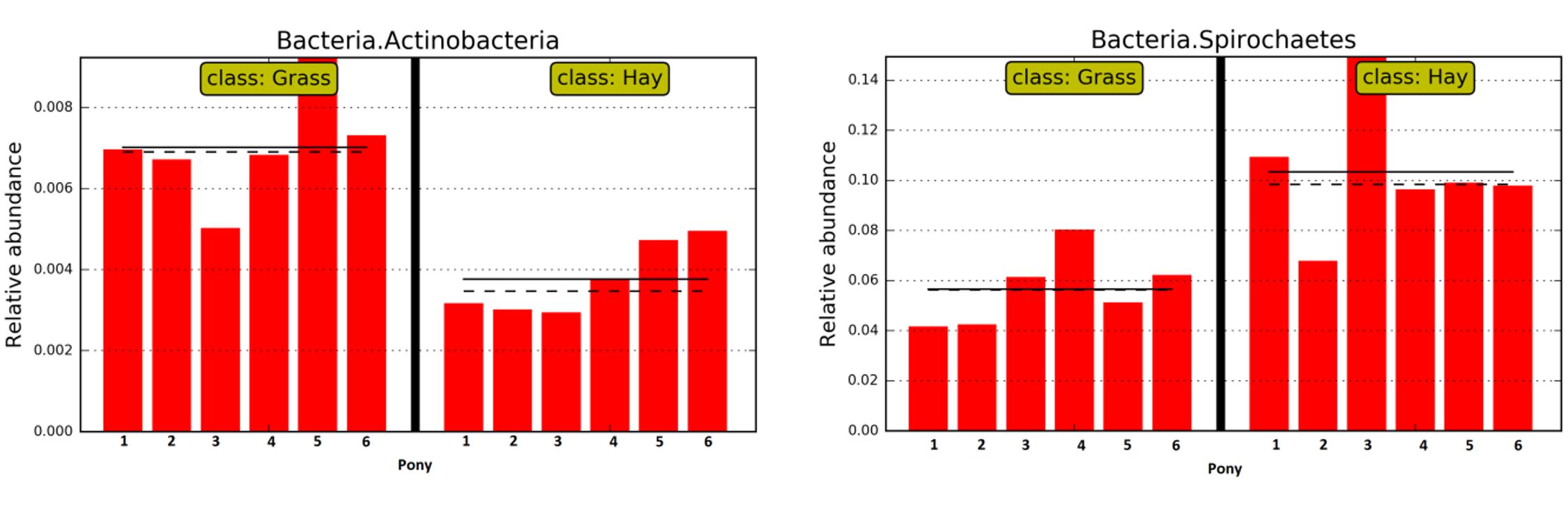

Supplement: S5 Fig — Solid and dashed horizontal lines indicate mean and median across all samples, respectively. (TIF) [file pone.0237869.s005.tif]

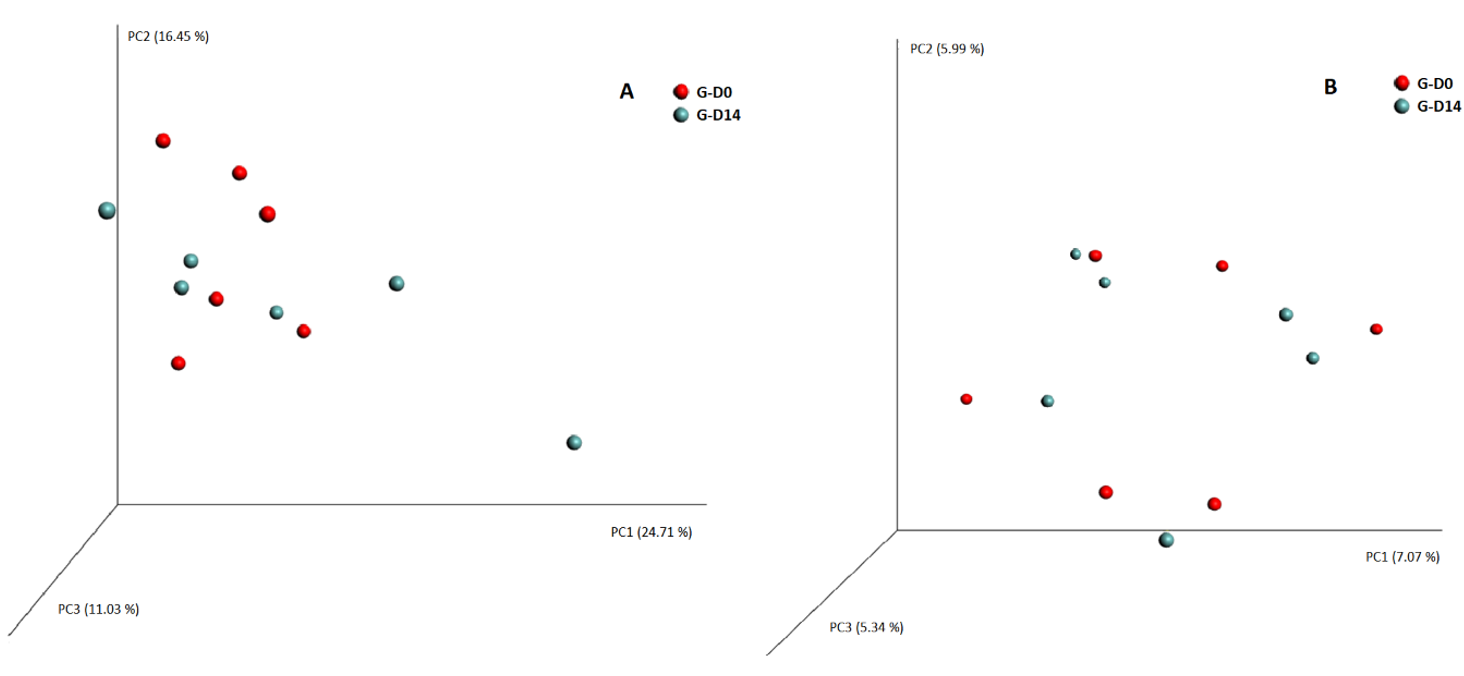

Supplement: S6 Fig — 3-dimensional Principal Coordinate Analyses plot (on a phylogenetic weighted (A) unweighted (B) UniFrac dissimilarity matrix) for data on the microbial membership and structure in the faeces of ponies (n = 6) across H-D14 and G-D14. There was no clustering observed. OTUs with <100 reads were excluded from the analysis. H: hay; G: grass; D: day. (TIF) [file pone.0237869.s006.tif]
