# Supplementary material for: Abrupt dietary changes between grass and hay alter faecal microbiota of ponies
Source: PLoS One. 2020 Aug 18;15(8):e0237869. doi: 10.1371/journal.pone.0237869 (PMC7446798; doi:10.1371/journal.pone.0237869)
Supplement: S1 Table — The bacterial and archaeal taxa identified in the faeces of ponies in the present study are listed in the table according to the taxonomic ranks using Greengenes database. (DOCX) [file pone.0237869.s007.docx]

| **Kingdom** | **Phylum** | **Class** | **Order** | **Family** | **Genus** |
| --- | --- | --- | --- | --- | --- |
| Bacteria | Bacteroidetes | Bacteroidea | Bacteroidales | unassigned | unassigned |
|  |  |  |  | unassigned | unassigned |
|  |  |  |  | BS11 | unassigned |
|  |  |  |  | Bacteroidaceae | BF311 |
|  |  |  |  |  | Bacteroides |
|  |  |  |  | Porphyromonadaceae | Paludibacter |
|  |  |  |  | Prevotellaceae | Prevotella |
|  |  |  |  | RF16 | unassigned |
|  |  |  |  | Rikenellaceae | unassigned |
|  |  |  |  |  | PW3 |
|  |  |  |  | S24-7 | unassigned |
|  |  |  |  | Paraprevotellaceae | unassigned |
|  |  |  |  |  | unassigned |
|  |  |  |  |  | CF231 |
|  |  |  |  |  | YRC22 |
|  |  |  |  |  | Prevotella |
| Bacteria | Firmicutes | Bacilli | Lactobacillales | Lactobacillaceae | Lactobacillus |
|  |  | Clostridia | Clostridiales | unassigned | unassigned |
|  |  |  |  | unassigned | unassigned |
|  |  |  |  | Christensenellaceae | unassigned |

| **Kingdom** | **Phylum** | **Class** | **Order** | **Family** | **Genus** |
| --- | --- | --- | --- | --- | --- |
|  |  |  |  | Clostridiaceae | unassigned |
|  |  |  |  |  | Alkaliphilus |
|  |  |  |  |  | Clostridium |
|  |  |  |  |  | Sarcina |
|  |  |  |  | Dehalobacteriaceae | unassigned |
|  |  |  |  |  | Dehalobacterium |
|  |  |  |  | Eubacteriaceae | Anaerofustis |
|  |  |  |  |  | Pseudoramibacter_ Eubacterium |
|  |  |  |  | Gracilibacteraceae | Lutispora |
|  |  |  |  | Lachnospiraceae | unassigned |
|  |  |  |  |  | unassigned |
|  |  |  |  |  | Anaerostipes |
|  |  |  |  |  | Blautia |
|  |  |  |  |  | Butyrivibrio |
|  |  |  |  |  | Coprococcus |
|  |  |  |  |  | Dorea |
|  |  |  |  |  | Epulopiscium |
|  |  |  |  |  | Lachnobacterium |
|  |  |  |  |  | Lachnospira |
|  |  |  |  |  | Roseburia |
|  |  |  |  |  | Shuttleworthia |
|  |  |  |  |  | Ruminococcus |
|  |  |  |  | Peptococcaceae | rc4-4 |
|  |  |  |  | Ruminococcaceae | unassigned |
|  |  |  |  |  | unassigned |
|  |  |  |  |  | Oscillospira |
|  |  |  |  |  | Ruminococcus |
|  |  |  |  | Syntrophomonadaceae | Syntrophomonas |
|  |  |  |  |  |  |
| **Kingdom** | **Phylum** | **Class** | **Order** | **Family** | **Genus** |
|  |  |  |  | Veillonellaceae | unassigned |
|  |  |  |  |  | Anaerovibrio |
|  |  |  |  |  | Phascolarctobacterium |
|  |  |  |  | Mogibacteriaceae | unassigned |
|  |  |  |  |  | Anaerovorax |
|  |  |  |  |  | Mogibacterium |
|  |  | Erysipelotrichi | Erysipelotrichales | Erysipelotrichaceae | unassigned |
|  |  |  |  |  | unassigned |
|  |  |  |  |  | Bulleidia |
|  |  |  |  |  | L7A_E11 |
|  |  |  |  |  | RFN20 |
|  |  |  |  |  | Eubacterium |
|  |  |  |  |  | p-75-a5 |
| Bacteria | Verrucomicrobia | Opitutae | Cerasicoccales | Cerasicoccaceae | unassigned |
|  |  | Verruco-5 | WCHB1-41 | RFP12 | unassigned |
|  |  |  |  | WCHB1-25 | unassigned |
|  |  | Verrucomicrobiae | Verrucomicrobiales | Verrucomicrobiaceae | Akkermansia |
|  |  | Pedosphaerae | Pedosphaerales | R4-41B | unassigned |
| Bacteria | Spirochaetes | MVP-15 | PL-11B10 | unassigned | unassigned |
|  |  | Spirochaetes | Sphaerochaetales | Sphaerochaetaceae | Sphaerochaeta |
|  |  |  | Spirochaetales | Spirochaetaceae | unassigned |
|  |  |  |  |  | Treponema |
| Bacteria | Fibrobacteres | Fibrobacteria | Fibrobacterales | Fibrobacteraceae | Fibrobacter |
| Bacteria | Actinobacteria | Actinobacteria | Actinomycetales | Microbacteriaceae | Agrococcus |
|  |  |  |  | Mycobacteriaceae | Mycobacterium |
|  |  | Coriobacteriia | Coriobacteriales | Coriobacteriaceae | unassigned |
|  |  |  |  |  | Adlercreutzia |
|  |  |  |  |  | Slackia |
| Bacteria | Proteobacteria | Alphaproteobacteria | unassigned | unassigned | unassigned |
| **Kingdom** | **Phylum** | **Class** | **Order** | **Family** | **Genus** |
|  |  |  | RF32 | unassigned | unassigned |
|  |  |  | Rickettsiales | unassigned | unassigned |
|  |  |  |  | mitochondria | unassigned |
|  |  | Betaproteobacteria | Burkholderiales | Alcaligenaceae | unassigned |
|  |  |  |  |  | Sutterella |
|  |  |  | Tremblayales | unassigned | unassigned |
|  |  | Deltaproteobacteria | Desulfovibrionales | Desulfovibrionaceae | unassigned |
|  |  |  |  |  | Bilophila |
|  |  |  |  |  | Desulfovibrio |
|  |  |  | GMD14H09 | unassigned | unassigned |
|  |  | Epsilonproteobacteria | Campylobacterales | Campylobacteraceae | Campylobacter |
| Bacteria | Tenericutes | Mollicutes | unassigned | unassigned | unassigned |
|  |  |  | Anaeroplasmatales | Anaeroplasmataceae | unassigned |
|  |  |  | Mycoplasmatales | Mycoplasmataceae | Anaeroplasma |
|  |  |  |  |  | unassigned |
|  |  |  | RF39 | unassigned | unassigned |
|  |  | RF3 | ML615J-28 | unassigned | unassigned |
| Bacteria | Cyanobacteria | 4C0d-2 | YS2 | unassigned | unassigned |
|  |  | Chloroplast | Streptophyta | unassigned | unassigned |
| Bacteria | Lentisphaerae | Lentisphaeria | Victivallales | Victivallaceae | unassigned |
|  |  |  | Z20 | R4-45B | unassigned |
| Bacteria | Planctomycetes | Planctomycetia | Pirellulales | Pirellulaceae | unassigned |
| Bacteria | TM7 | TM7-3 | CW040 | F16 | unassigned |
| Bacteria | Synergistetes | Synergistia | Synergistales | Synergistaceae | unassigned |
| Bacteria | Elusimicrobia | Elusimicrobia | Elusimicrobiales | Elusimicrobiaceae | Elusimicrobium |
|  |  |  |  |  | unassigned |
|  |  | Endomicrobia | unassigned | unassigned | unassigned |
| Bacteria | Chloroflexi | Anaerolineae | Anaerolineales | Anaerolinaceae | SHD-231 |
|  |  |  |  |  |  |
| **Kingdom** | **Phylum** | **Class** | **Order** | **Family** | **Genus** |
| Bacteria | WPS-2 | unassigned | unassigned | unassigned | unassigned |
| Bacteria | SR1 | unassigned | unassigned | unassigned | unassigned |
| Bacteria | Unassigned | unassigned | unassigned | unassigned | unassigned |
| Bacteria | Unassigned; Other | other |  |  |  |
| Archaea | Euryarchaeota | Methanobacteria | Methanobacteriales | Methanobacteriaceae | Methanobrevibacter |
|  |  | Methanomicrobia | Methanomicrobiales | Methanocorpusculaceae | Methanocorpusculum |
|  |  |  | Methanosarcinales | Methanosarcinaceae | Methanimicrococcus |
|  |  | Thermoplasmata | E2 | Methanomassiliicoccaceae | unassigned |
|  |  |  |  |  | vadinCA11 |
